# A low-profile broadband crossed-dipole antenna with fractal structure and inverted-L plates

**DOI:** 10.1038/s41598-022-18594-x

**Published:** 2022-09-02

**Authors:** Guirong Feng, Tianyi Qi, Mingming Li, Jiachen Li, Xin-wei Wang, Hai-long Yang

**Affiliations:** 1 School of Electrical Engineering, Xi’an University of Posts and Telecommunications, Xi’an, 710100 China; 2 School of Electrical Engineering, Xi’an University of Posts and Telecommunications, Xi’an, 710121 China

**Keywords:** Electrical and electronic engineering, Mechanical engineering

## Abstract

A low-profile broadband circularly polarized (CP) crossed-dipole antenna is proposed. In this antenna, the dipole uses a fractal-based structure with multiple similar patches for obtaining broadband CP radiation and impedance matching. The incorporation of the crossed-dipole antenna with four triangular parasitic patches improves both the impedance and axial ratio (AR) bandwidths. Four parasitic coupled inverted-L metal plates are loaded on the ground plane to significantly enhance the AR bandwidth and reduce the antenna profile. The measured results are in agreement with the simulations, which demonstrates that the proposed antenna has a low profile of 0.18 λ_0_, a − 10 dB impedance bandwidth of 91% (2.78–7.42 GHz), a 3 dB AR bandwidth of 81.5% (2.99–7.1 GHz), and a good right hand circular polarization (RHCP) radiation pattern with an average gain of 7.9 dBi over the whole operating band.

## Introduction

Circularly polarized (CP) antennas have been widely used in various wireless communication systems, because they have superior advantages over linearly polarized (LP) antennas, such as mitigating polarization mismatch, combating multipath interference, and providing stable link between transmitting and receiving antennas. As a very important antenna type, a single-fed CP crossed-dipole antenna with a 90° phase-shift ring has attracted much attention due to its simple structure and wide bandwidth^1,2^. However, such crossed-dipole antenna has a relative narrow axial ratio (AR) bandwidth of less than 30% and a high profile of about 0.25 λ_0_^2^. In recent years, broadband CP antennas have gained great popularity in high-speed-rate communications. In addition, CP antennas with a low profile are very essential for conformal carriers or space-constrained platforms. Therefore, it is important to design a broadband and low-profile CP crossed-dipole antenna for modern wireless communication systems.

To realize a broadband CP crossed-dipole antenna, one method is to use wideband dipoles^3–6^, which can provide two orthogonal fields with equal amplitude in a wide band for generating broadband CP radiation. For example, the antenna using rectangular dipole and bowtie dipole can achieve AR bandwidths of 27%^3^ and 43%^4^, while using L-shape dipole and elliptical dipole can increase the bandwidths to 67.5%^5^ and 96.6%^6^, respectively. Moreover, the combination of wideband dipole and parasitic elements can further enhance the bandwidths^7–9^. In particular, this method of adding four parasitic elements in a sequential rotation way can achieve a fairly wide CP bandwidth^10–12^. For example, a crossed bowtie dipole antenna integrating with three groups of four parasitic elements obtains a bandwidth of 90.9%^11^, while four vertical metal plates were added on the ground plane of a rectangular dipole antenna, obtaining a wide bandwidth of 106.1%^12^. Although the aforementioned antennas can achieve a wide CP bandwidth, their profiles are still as high as 0.25 λ_0_.

To reduce the profile of the crossed-dipole antenna, an artificial magnetic conductor (AMC) can be used instead of a metal ground plane^13–15^. For example, the AMC ground in^15^ reduces the antenna profile to 0.16 λ_0_, but giving two narrow AR bandwidths of 19.3% and 33.8%. In addition, low profile can also be realized by introducing shorted elements into crossed-dipole antennas^16–20^. In^20^, the antenna with a profile of 0.1 λ_0_ achieves an AR bandwidth of 63.4% by adding four shorted parasitic patches on the bottom side of the substrate. Similarly, by adding two groups of shorted parasitic patches on the both sides of substrate, a much lower profile of 0.067 λ_0_ were obtained but at a cost of modest operating bandwidth of 51.6%^21^. Therefore, it is still a big challenge to design a CP crossed-dipole antenna with both wide bandwidth and low profile simultaneously.

In this paper, without increasing extra radiators, a CP crossed-dipole antenna uses a fractal-based structure to design the dipole for obtaining a very wide impedance bandwidth. In addition, four parasitic patches are loaded around the crossed-dipole to increase the CP bandwidth. Four coupling parasitic inverted-L plates are shorted to the ground plane to enhance the AR bandwidth and reduce the antenna profile. Specially, the coupled shorted plates instead of direct shorting the parasitic elements^20^ to grounds wouldn`t cause large back radiation of cross-polarization when the antenna has a low profile. It has been found that the antenna with a low profile of 0.18 λ_0_ achieves a wide impedance bandwidth of 91% and AR bandwidth of 81.5%.

## Antenna design

### Antenna geometry

**Figure 1:**
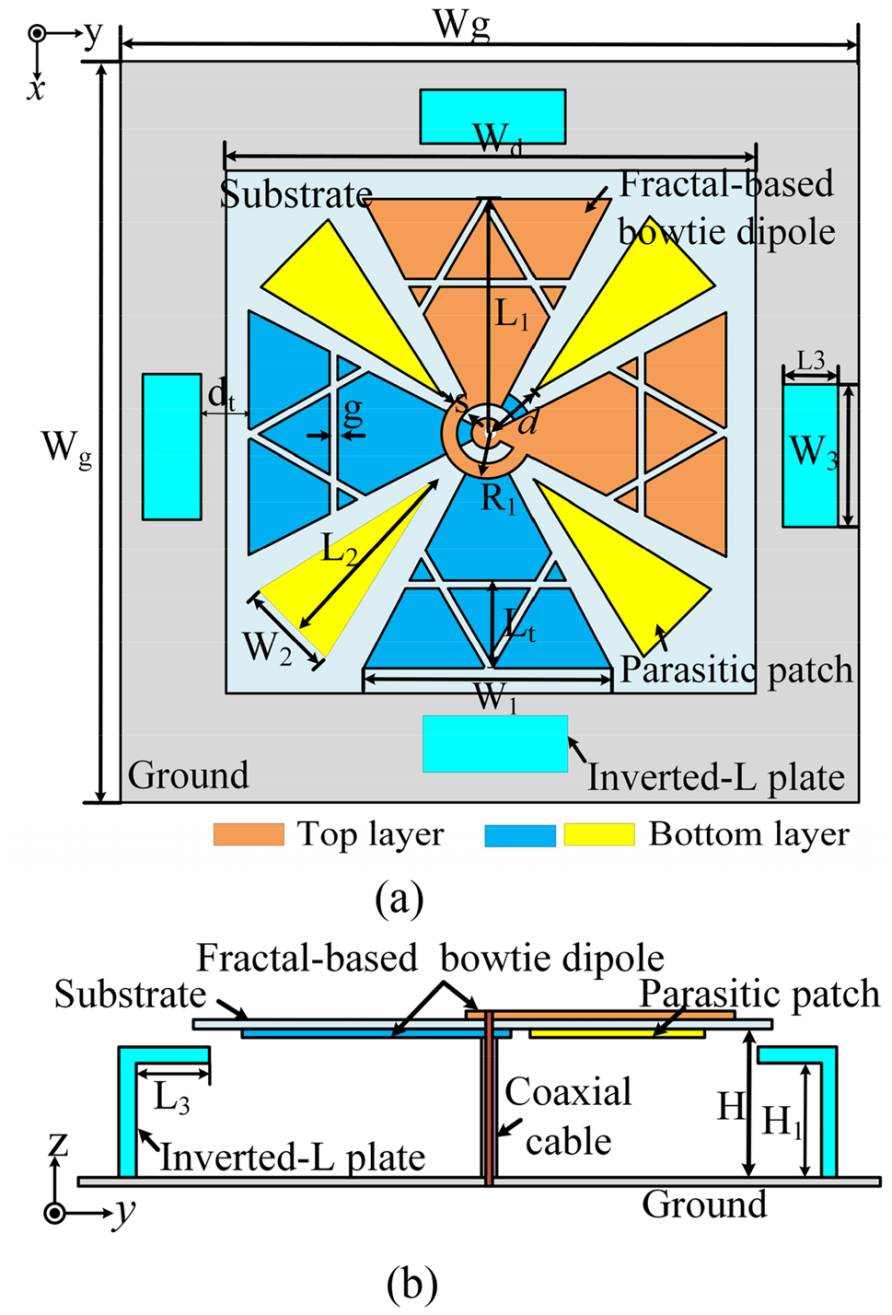
Configuration of the proposed antenna. (**a**) Top view of the antenna; (**b**) side view of the antenna. Wg = 74.5 mm, Wd = 40 mm, L1 = 19.2 mm, W1 = 20.5 mm, R1 = 1.93 mm, s = 0.34 mm, Lt = 8.56 mm, g = 0.5 mm, L2 = 16.9 mm, W2 = 8 mm, d = 4.6 mm, dt = 4 mm, W3 = 10 mm, L3 = 5.5 mm, H1 = 9.3 mm, H = 10.8 mm.

Figure 1 shows the geometry of the proposed low-profile broadband CP crossed-dipole antenna, which is printed on a 0.5-mm-thick substrate with a relative permittivity of 2.65. The antenna consists of two pairs of crossed dipoles, four triangular parasitic patches, four parasitic inverted-L metal plates, a square ground plane, and a coaxial cable. The two pairs of crossed-dipoles are etched on both sides of the substrate and soldered to the inner and outer conductors of the coaxial cable, respectively. To generate CP radiation, two perpendicular dipoles are connected by a 90° phase-shift ring with a circumference of about λ_0_/4. In this antenna, each dipole uses a fractal-based structure, which can be designed by cutting three slits with a width of *g* from a bowtie-dipole to yield several similar patches without increasing extra radiators and size. To enhance the bandwidths, four triangular patches are loaded on the bottom side of the substrate, which are rotationally placed along the diagonal for exciting additional CP resonance. In addition, four parasitic inverted-L plates, consisting of a horizontal rectangular plate and a vertical plate with a height of H1 below the substrate, are rotationally loaded on the ground plane to enhance the operating bandwidth. Besides, the parasitic plates can also help to reduce the antenna profile by properly tuning the coupling between the parasitic plate and crossed-dipoles. The square ground plane is located below the substrate by a distance of H for providing a unidirectional radiation pattern. The optimized parameters of the proposed antenna are listed in the caption of Fig. 1.

### Working mechanism

To illustrate the design process and bandwidth enhancement, three reference antennas are compared with the proposed antenna. Reference Antenna I is a simple crossed bowtie dipole antenna; Reference Antenna II modifies the bowtie-dipole by using a fractal-based structure; In Reference Antenna III, the fractal-based dipoles are used and four triangular parasitic patches are loaded. For a fair comparison, the dimensions of these reference antennas are the same with the proposed antenna.

**Figure 2:**
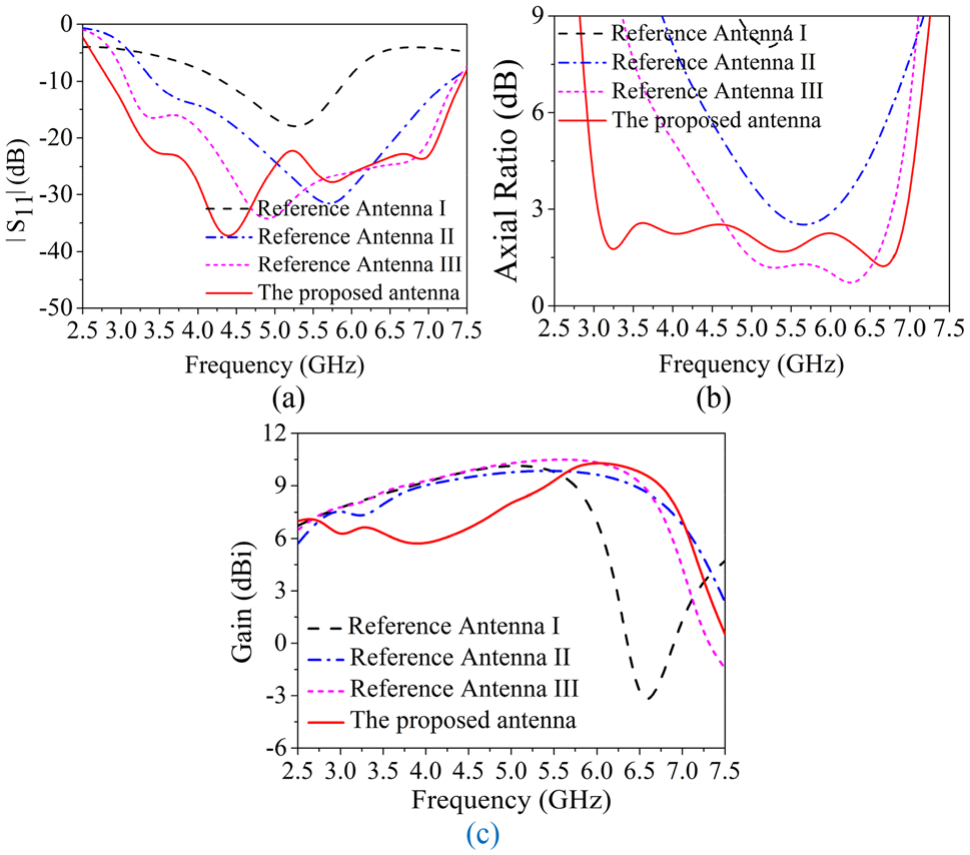
Simulated reflection coefficients, ARs and gain of the antennas. (**a**) Reflection coefficients, (**b**) ARs, and (**c**) gain.

**Figure 3:**
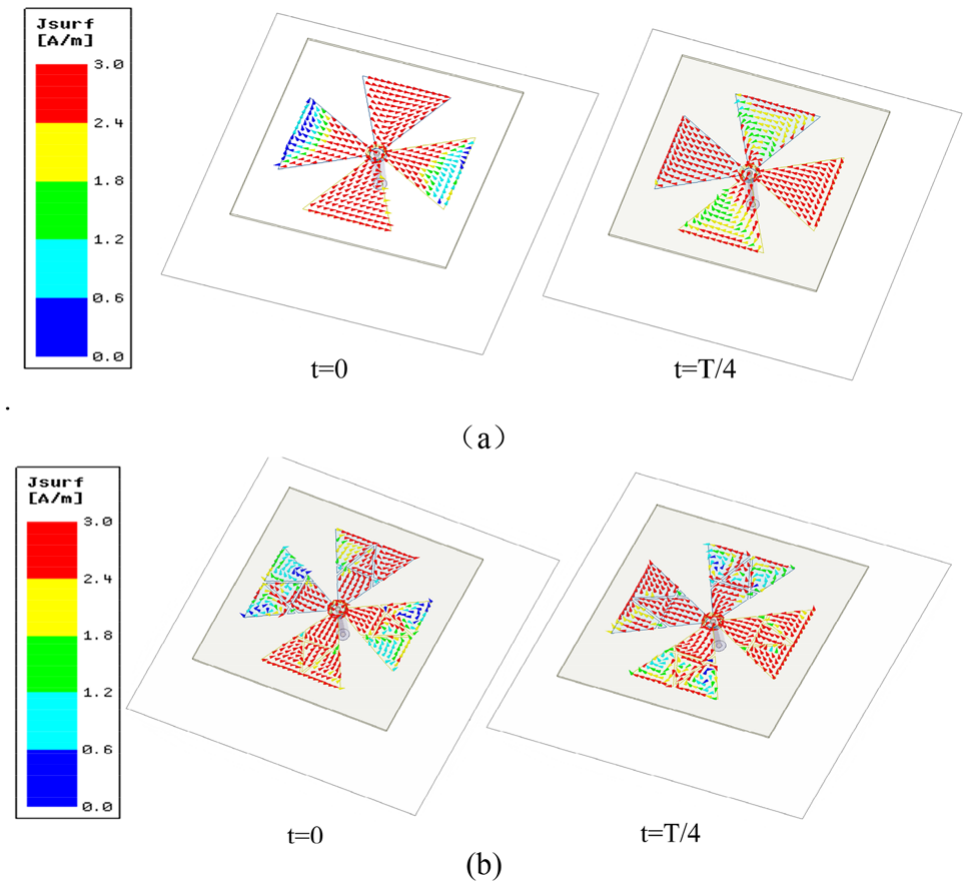
Current distribution on the crossed dipole of reference antennas. (**a**) Reference antenna I, (**b**) reference antenna II.

**Figure 4:**
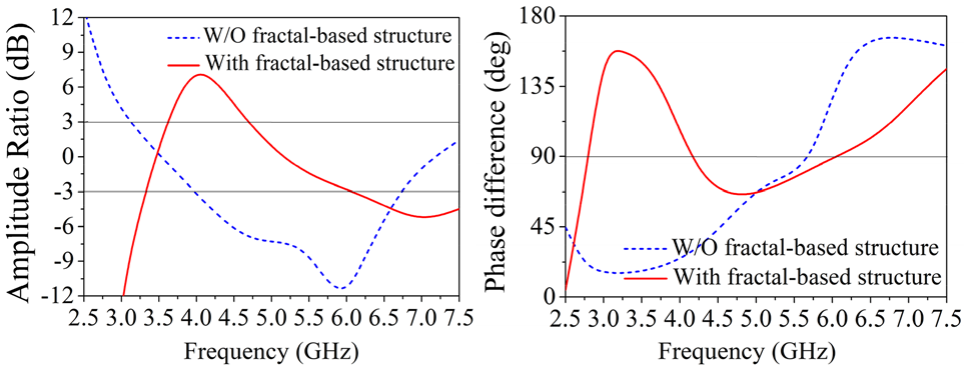
Amplitude ratio and phase difference of two orthogonal modes in reference antenna II.

**Figure 5:**
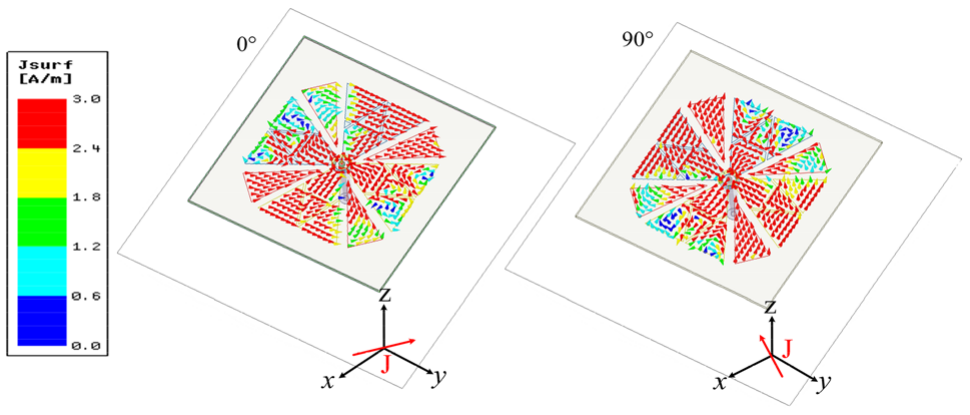
Current distribution on the parasitic patches of antenna 3 at 6.25 GHz.

The simulated refection coefficient, AR and boresight gain of these antennas are shown in Fig. 2. As depicted, Antenna I has a relative narrow impedance bandwidth (43.3%) and poor AR because the antenna height is only 0.18 λ_0_ but not λ_0_/4. While the impedance bandwidth of Reference Antenna II is significantly enhanced by using fractal-based bowtie dipoles. Since the proposed fractal-based dipole consists of multiple similar patches operating at different operating frequency, and the combined bands can increase the overall bandwidth^22^. To investigate this modified dipole, current distributions of crossed-dipole on reference antenna I and II are examined and shown in Fig. 3. From Fig. 3a, it can be seen that the current amplitude on Antenna I at t = 0 and t = T/4 varies greatly, while the fractal-based structure can balance the current amplitude to generate one more operating band (Fig. 3b). As a result, Antenna II achieves wideband characteristic. For better understanding the role of fractal-based dipole on AR, the amplitude ratio and phase difference of two orthogonal modes are investigated in Fig. 4. As can be seen from the figure, it is easier for Antenna II than Antenna I to generate CP radiation when the amplitude ratio of two orthogonal modes in range of − 3 to 3 dB and their phase difference around 90° are simultaneously satisfied in a same band. Hence, the AR of Antenna II is better than that of Antenna I. When introducing four triangular parasitic patches in Reference Antenna III, the impedance matching in the low band is improved because of the parasitic loading effect. Moreover, the CP mode of crossed-dipole shifts downward to 5.25 GHz. By coupling with the crossed-dipoles, these parasitic patches achieve a sequential rotated phase distribution, so a new CP resonance at 6.25 GHz is excited to enhance the AR bandwidth, the current distribution at this frequency can be seen from Fig. 5.

When the four parasitic inverted-L plates are introduced to the proposed antenna, S_11_ shifts to lower band, which suggests that the current path could be lengthened, as a result the antenna profile can be reduced. On the other hand, a very wide impedance bandwidth is obtained. Also, an extra CP resonance mode at 3.25 GHz is generated owing to the coupling between the inverted-L plates and the crossed-dipoles, hence the AR bandwidth is substantially broadened.

**Figure 6:**
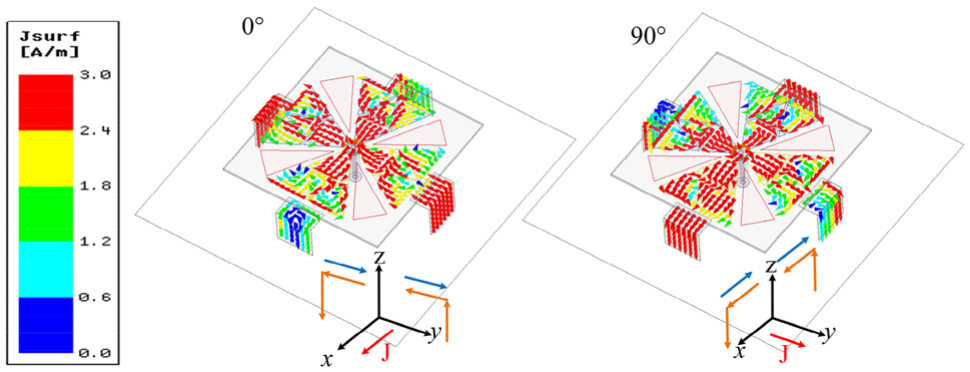
Current distribution on the crossed dipoles and inverted-L plates of the proposed antenna at 3.25 GHz. The blue line represents the current vector on the crossed-dipole. The orange line represents the current vector on the inverted-L plate.

To illustrate the working mechanism of the proposed antenna, the current distributions on the crossed-dipole and inverted-L plates at 3.25 GHz are shown in Fig. 6. As is observed, strong currents concentrate on the plates owing to the strengthened coupling with the crossed-dipole. At phase of 0°, the horizontal *y*-direction currents on the crossed-dipole and inverted-L plates flow in the opposite direction and can cancel each other; While the vertical *z*-direction currents on two opposite inverted-L plates can also be cancelled. Therefore, only the horizontal *x*-direction currents on crossed-dipoles dominate the total current *J*. Similarly, at phase of 90°, the total current is dominated by the horizontal *y*-direction currents on the crossed-dipole. It is seen that the two total currents are orthogonal to each other and their current intensity are comparable. Therefore, an additional CP mode is generated and a good right-hand circular polarization (RHCP) pattern is obtained. On the other hand, these coupled shorted plates can balance the amplitude of crossed dipole and parasitic patches to broaden the bandwidth.

In addition, it can also be observed that the currents on the dipoles are coupled to the plates and finally shorted to the ground plane. Therefore, the inverted-L plates can be regarded as shorting walls to some extent, which will increase the current path of crossed-dipole and reduce the antenna profile. On the other hand, the vertical *z*-directional current on the plates is similar to that of a monopole antenna, providing a monopole-like radiation pattern. As well known, the crossed-dipole antenna radiates a heart-shape pattern in the boresight direction. When introducing the coupling shorted plates into the crossed-dipole antenna, their radiation patterns are superimposed with each other, generating a heart-shape radiation pattern but with wide beam width^18,20^. However, since the monopole-like radiation pattern exhibits a deep null in the broadside, therefore the superimposed pattern has a lower gain in 3.5**–**4 GHz, this explains the gain descend in the lower band as shown in Fig. 2c.

### Parameter study

**Figure 7:**
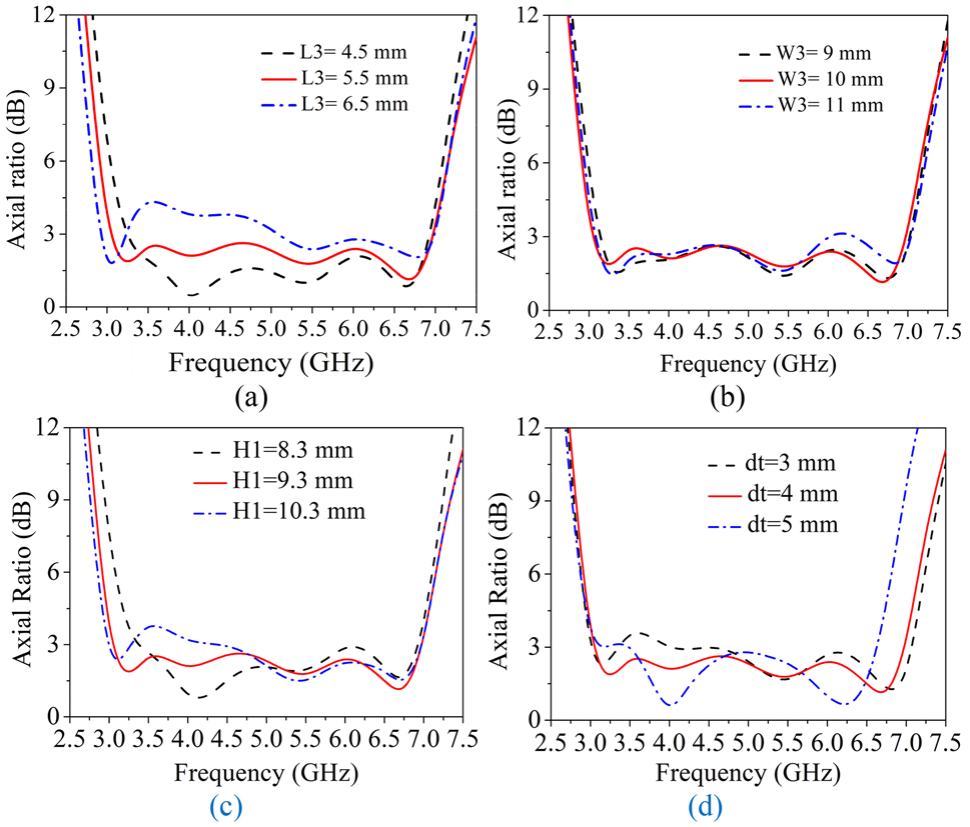
Simulated ARs for different parameters. (**a**) L3, (**b**) W3, (**c**) H1, and (**d**) dt.

To further investigate the effect of coupling between the crossed-dipole and inverted-L plates on AR bandwidth, the plate size (W3, L3, H1) and the distance (dt) from the crossed-dipole are studied in Fig. 7. With reference to the figure, it can be seen that as the parameter L3 increases the AR points shift downward, while the lower band AR gradually gets worse. In addition, the AR is not sensitive to the parameter W3 which has slight effect on the upper band and the whole AR bandwidth. While the parameter H1 dramatically affects the lower band AR response, therefore it is necessary to properly adjust the height of inverted-L plates to achieve wide bandwidth, because as H1 varies, the capacitance to the inverted-L plate from the dipole follows. When the parameter dt increases, the upper band AR points shifts to lower band and the lower band AR has a minor deterioration. Obviously, the parameter dt plays a crucial role on the AR bandwidth.

## Experimental result

**Figure 8:**
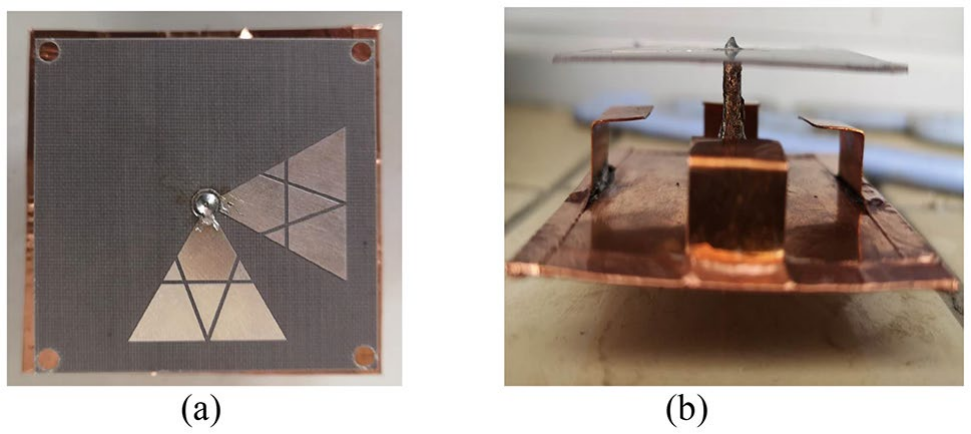
Prototype of the proposed antenna. (**a**) Top view, (**b**) side view.

**Figure 9:**
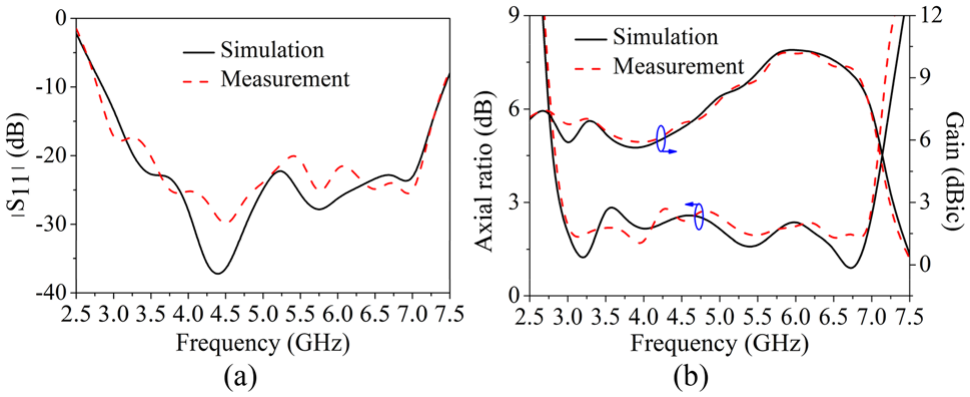
Simulated and measured reflection coefficients, ARs and gain of the prototype. (**a**) Reflection coefficients. (**b**) ARs and boresight gains.

**Figure 10:**
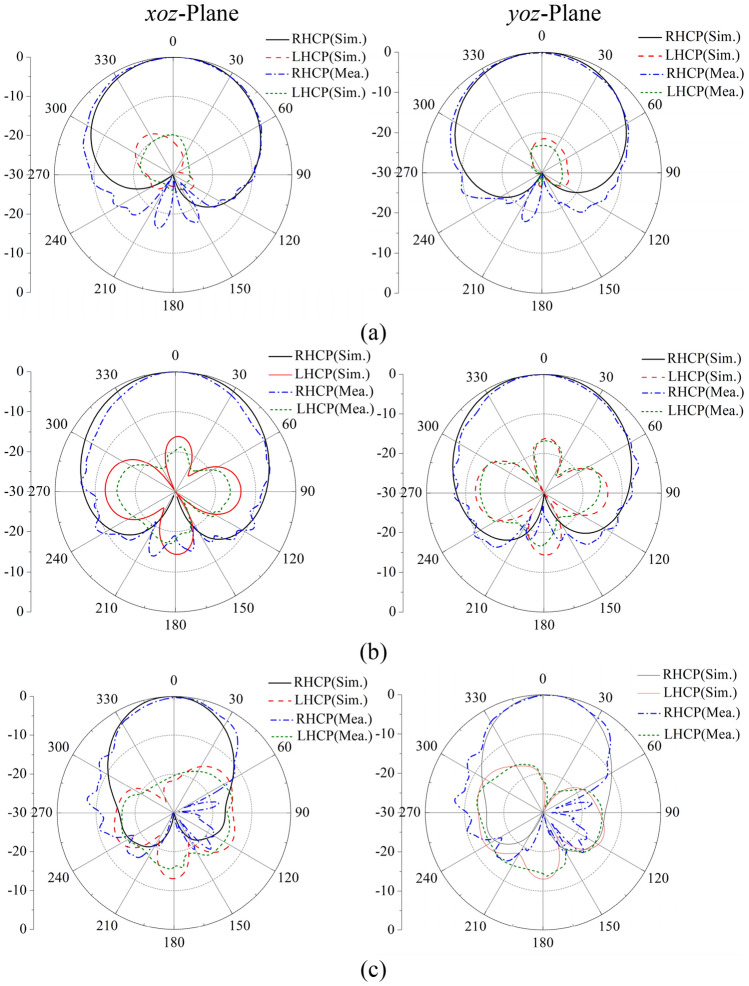
Simulated and measured radiation patterns of the prototype. (**a**) 3.3 GHz. (**b**) 4 GHz. (**c**) 6.7 GHz.

To validate the proposed design, a prototype was fabricated, as shown in Fig. 8. The simulated and measured reflection coefficients, ARs and boresight gains are shown in Fig. 9. A reasonable agreement between the simulated and measured results can be observed. The simulated and measured impedance bandwidth are given by 89% (2.86**–**7.45 GHz) and 91% (2.78**–**7.42 GHz); the simulated and measured AR bandwidths are 82.4% (2.97**–**7.13 GHz) and 81.5% (2.99**–**7.1 GHz), respectively. While the measured average gain is 7.9 dBi within the operating band. Figure 10 shows the simulated and measured radiation patterns at 3.3, 4, and 6.7 GHz. The RHCP patterns at these frequencies are all higher than left-hand CP (LHCP) by 18 dB, demonstrating the antenna generates RHCP radiation pattern. It can be seen that the beam-width of the pattern at 4 GHz is much wider than that at other frequencies. This is because the vertical part of the inverted-L plate has a great effect. In addition, by the merit of using the coupling shorted parasitic plates instead of direct shorting the parasitic elements to grounds in^20,21^, lower back radiations of cross-polarization are obtained.

A comparison between the proposed and previous CP crossed-dipole antennas are tabulated in Table 1. As seen from the table, the proposed antenna has a competitive bandwidth (impedance, AR and overlapping bandwidths), and the antenna profile is relatively low. In addition, the proposed antenna has a highest broadside gain. Although the antenna presented in^12^ has rather wide bandwidths, and the antennas in^19^ and^20^ has a very low profile, the back radiation of the cross-polarized fields are very large. While the other antennas have either comparative profile or narrow bandwidths.

**Table 1:** Comparison between the proposed and the previously reported crossed-dipole antennas.

	Overall size (λ_0_)	IBW (%)	ARBW (%)	Peak gain (dBi)	Back radiation of cross-polarization (dB)
^9^	0.6 × 0.6 × 0.21	98.2	85.5	10	–
^10^	0.29 × 0.29 × 0.1	63.2	52.4	–	–
^11^	0.4 × 0.4 × 0.21	93.1	90.9	8.6	–
^12^	0.28 × 0.28 × 0.11 × 0.11	115.2	106.2	7	> − 10
^15^	0.54 × 0.54 × 0.13	40/49.5	19.3/33.8	6.6/7.4	–
^19^	–	< 90	< 59	< 9	–
^20^	0.31 × 0.31 × 0.07	78.3	63.4	< 5	> − 10
^21^	0.32 × 0.32 × 0.04	61.8	51.6	< 5	> − 5
Proposed	0.75 × 0.75 × 0.11	91	81.5	10.3	< − 10

λ_0_: Wavelength at the lowest frequency of passband.

^19^: An antenna element.

## Conclusion

A low-profile broadband CP crossed-dipole antenna is studied. A fractal-based structure is applied to the bowtie dipole to generate wide CP radiation, and parasitic patches are used to further broaden the AR bandwidth. The inverted-L plates can only excite addition CP mode to enhance the AR bandwidth but also reduce the antenna profile. A prototype has been fabricated and measured. The measured results show that the prototype has a low profile of 0.13 λ_0_, impedance bandwidth of 91%, and AR bandwidth of 81.5%.

## Data Availability

The datasets used and/or analyzed during the current study available from the corresponding author on reasonable request.
